# Epidemiological situation of bovine and bubaline tuberculosis in the state of Pará, Amazon region of Brazil

**DOI:** 10.3389/fvets.2024.1466199

**Published:** 2024-11-20

**Authors:** Bruno Cesar Ribeiro da Silva Oliveira, Jefferson Pinto Oliveira, Ana Paula Vilhena Beckman Pinho, Ricardo Augusto Dias, José Henrique Hidebrand Grisi Filho, Vitor Salvador Picão Gonçalves, Marcos Bryan Heinemann, Marcos Amaku, Evelise Oliveira Telles, Bruno Fontana Soares Ferreira, Fernando Ferreira, José Soares Ferreira Neto

**Affiliations:** ^1^Faculty of Veterinary Medicine and Animal Science, University of São Paulo, São Paulo, Brazil; ^2^Agência de Defesa Agropecuária do estado do Pará, Belém, Brazil; ^3^WOAH-Collaborating Centre for Economics of Animal Heath in the Americas Region, São Paulo, Brazil; ^4^Faculty of Agronomy and Veterinary Medicine, University of Brasilia, Brasilia, Brazil

**Keywords:** bovine tuberculosis, bubaline tuberculosis, prevalence, risk factors, control, Pará, Brazil

## Abstract

**Introduction:**

Bovine tuberculosis is one of the primary infectious diseases affecting cattle. Although several countries have managed to eradicate this zoonosis it remains endemic and uncontrolled across many countries in Africa, Asia, Latin America, and the Middle East. Brazil launched its national control and eradication program in 2001, and since then, epidemiological studies have been carried out to define optimal control strategies and to enable the management of the process in each region.

**Methods:**

This study covered the state of Pará, which was divided into three regions, in each of which a pre-established number of properties were randomly selected, and within each property, a minimum number of animals were drawn to be tested by the tuberculin test to classify the farm as infected or free of the disease. A questionnaire was administered to the selected properties to identify the risk factors for the disease.

**Results:**

A total of 976 properties comprising 17,151 animals were tested. The prevalence of infected properties in the regions ranged from 3.1% [1.3; 4.9] to 18.6% [14.3; 22.9], while tuberculin-positive animals ranged from 0.24% [0.09; 0.40] to 4.8% [2.4; 7.3]. The introduction of cattle untested for bTB and the renting of pastures have been identified as risk factors for the disease in this state.

**Discussion:**

Pará has one of the highest prevalences of bovine tuberculosis in Brazil, and the implications of these results for public health were discussed. We therefore proposed that the state’s Official Veterinary Service, together with the private dairy and meat sectors, pursue the objective of controlling or eradicating the disease, along with mechanisms to verify its effectiveness. The differences between the two objectives were discussed, but in both cases, an education program is necessary to inform cattle and buffalo breeders that they need to test animals for tuberculosis before introducing them to their farms, and also to avoid renting pastures for cattle to graze and rest while moving on foot, as these practices have contributed the most to the spread of bTB in the state.

## 1 Introduction

Bovine and buffalo tuberculosis (bTB) is a chronic zoonotic disease caused by bacteria of the *Mycobacterium tuberculosis* complex, predominantly *Mycobacterium bovis*, as reported by the World Organization for Animal Health. It is capable of infecting many domestic and wild animals, but is one of the primary infectious diseases in cattle, causing impaired general health, pneumonia, weight loss, and, in rare cases, death (1). Cattle are the main source of *M. bovis* infections in animals and humans (1). In regions with a high prevalence of human tuberculosis and close interfaces with cattle, the transmission of *M. tuberculosis*, *M. bovis* and *Mycobacterium orygis* from humans to animals has been recorded (1).

Several species of wild animals have been identified as reservoirs of the disease resulting in the infection of domestic cattle in Canada (*Cervus canadensis*, Elk), the Iberian Peninsula (*Sus scrofa* (Wild boar) and some cervids), Ireland and the United Kingdom (*Meles meles*, Badger), New Zealand (*Trichosurus vulpecula*, Brushtail possum), South Africa (*Syncerus caffer*, African buffalo), and certain regions of the United States (*Odocoileus virginianus*, White-tailed deer) (1).

A country can declare itself free of bTB by demonstrating through a surveillance system that, in the last 3 years, 99.8% of the premises were tested and found to be uninfected (2). Only Australia, the Netherlands, Sweden, Denmark, Finland, Estonia, the Czech Republic, Latvia, Lithuania, Luxembourg, Slovakia, and Canada are considered officially bTB-free (OTF) with no cases reported in the last 3 years; whereas, France, Germany, Austria, Poland, Belgium, Hungary, and Slovenia are also OTF, but with a few cases reported annually (3). In 2021, Japan declared itself free of *M. tuberculosis* complex infections in cattle, while Egypt declared the same for its dairy cattle compartment (4). In 2023, Namibia declared its Foot-and-Mouth Disease-free zone to be free of *M. tuberculosis* complex infections in cattle (4). However, bTB remains endemic and uncontrolled in much of Africa, Asia, Latin America, and most Middle Eastern countries (1, 5).

In Brazil, the Ministry of Agriculture, Livestock and Food Supply (MAPA) launched the National Program for the Control and Eradication of Brucellosis and Tuberculosis (PNCEBT) in 2001 (6), which covers cattle and buffalo and is based on mandatory testing before moving animals, compulsory slaughter of positive ones, and voluntary adherence to the accreditation process for farms free of tuberculosis and brucellosis. Since then, several standardized epidemiological studies have been conducted in Brazilian states to estimate the prevalence of bTB-infected farms and comparative cervical tuberculin-positive animals with the aim of choosing the best strategies based on each region’s individual situation, and allowing the effectiveness of actions to be verified in view of the initial prevalence. The different states can choose which strategies to use, either control or eradication. The only states that are implementing eradication strategies are Mato Grosso and Santa Catarina.

These standardized studies have been coordinated by MAPA and the Official Veterinary Service of the states, with scientific support provided by our Collaborating Center for Animal Health at the Faculty of Veterinary Medicine and Animal Science (FMVZ) of the University of São Paulo (USP). To date, 14 of Brazil’s 27 states, covering 77% of the country’s cattle and buffalo population, have been studied.

**Figure 1:**
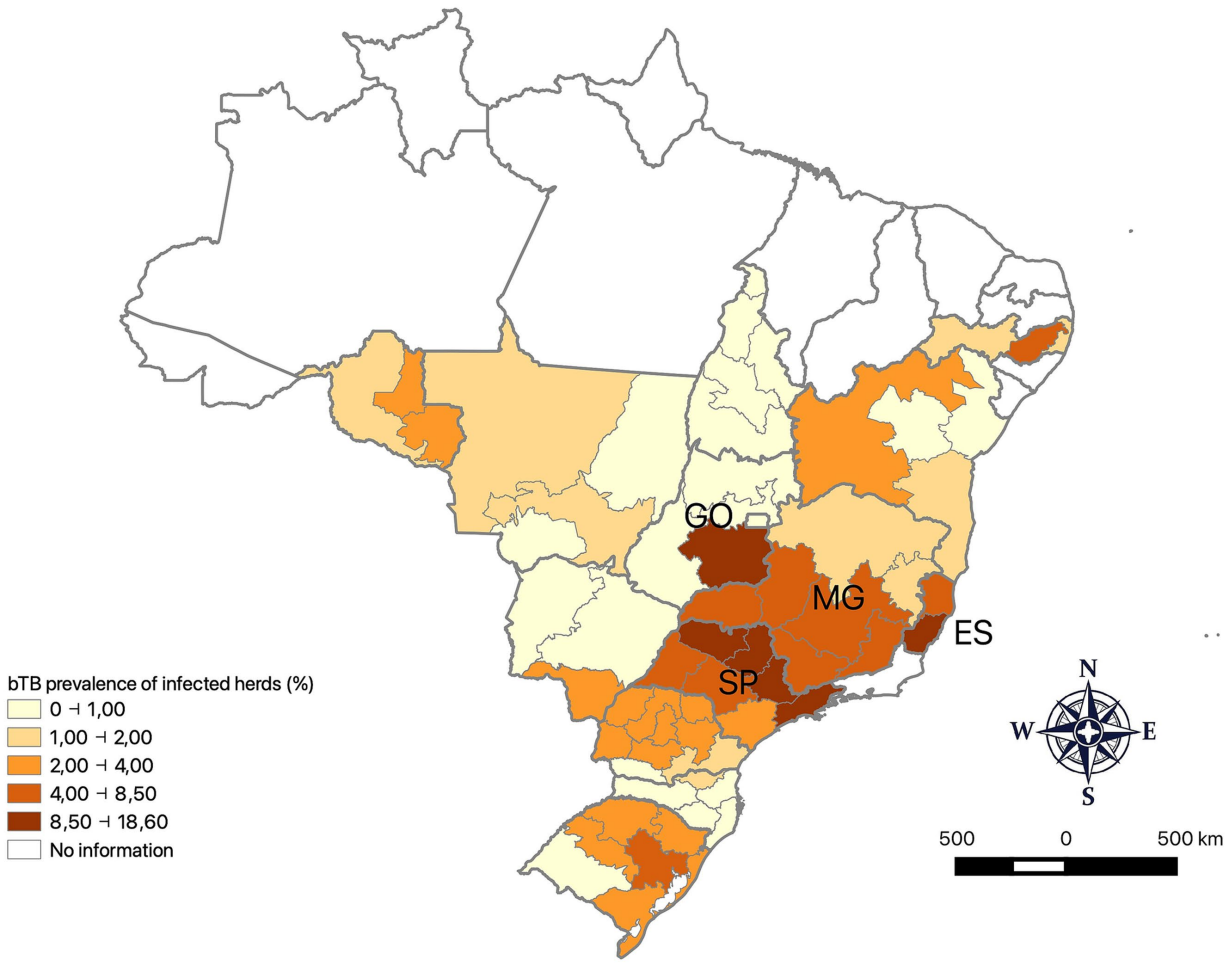
Prevalence of bTB-infected herds in Brazil, showing a region of high prevalence covering the entire state of Espírito Santo (ES), South of Minas Gerais (MG), North of São Paulo (SP) and Southeast of Goiás (GO). Source: Ferreira Neto et al. (9) and Rodrigues et al. (3).

These studies have shown that there are major differences in the prevalence of bTB-infected herds between states (Figure 1), with extremes of 0.16 and 9.0% in the states of Tocantins and São Paulo, respectively (7, 8). Furthermore, bTB in Brazil is predominantly associated with dairy farms, especially the high-performance ones, as well as with the introduction of animals not tested for tuberculosis on farms (9).

The highest prevalence areas have a large concentration of high production dairy farms, characterized by European breeds in intensive and semi-intensive systems, with mechanical milking and use of artificial insemination. Unlike the extensive system used in the vast majority of cattle and buffalo farms in Brazil, on these farms, the high animal density and the greater concentration of cows for milking increases the risk of *M. bovis* transmission. The lower prevalences in other regions of Brazil are mainly explained by the absolute predominance of beef or mixed properties operating under an extensive production system.

Pará is the second largest Brazilian state, covering 1.2 million km^2^, and housing 8.1 million inhabitants and 25 million cattle, representing approximately 10% of the national cattle population (10, 11). The state has 41% of the 1.6 million domestic buffalo in Brazil (11). Around 66% of the 650,000 buffalo in the state of Pará are located in the Marajó archipelago, where they are raised extensively as working animals, as well as for the production of meat, milk, and leather in lowland and mangrove areas, adapting to the seasonal and climatic variations governed by flood and ebb regimes, which are peculiar to the region (12).

The data available on the epidemiological situation of bovine and bubaline tuberculosis in the state of Pará comes from regional studies with convenience samples, and does not allow the results to be extrapolated to the entire population of the state. However, it can be stated that the disease is endemic and affects both species (13–18). Although the PNCEBT began in 2001, there are currently no farms accredited as bTB-free in the state. Thus, the objectives of this study were to identify the risk factors for bovine and bubaline tuberculosis in the state of Pará, to estimate the prevalence of bTB at farm and animal level, and to verify the existence of heterogeneities between regions through a cross-sectional study. The generated data will inform future decision making regarding combating the disease in the state.

## 2 Methods

This study was planned by MAPA and the Official Veterinary Service (OVS) of the state of Pará (Agência de Defesa Agropecuária do Estado do Pará—ADEPARÁ), with scientific support provided by the Collaborating Center for Animal Health at the Faculty of Veterinary Medicine and Animal Science (FMVZ) of the University of São Paulo (USP). The fieldwork was carried out by ADEPARÁ between 2018 and 2020.

### 2.1 Study design

To capture possible internal heterogeneities, the state of Pará was divided into regions, based on animal production and marketing systems, management practices, and cattle and buffalo farming enterprise (beef, dairy or mixed); in addition, ADEPARÁ’s operational and logistical capacity was also considered. No objective data from the OVS property register was used, only the knowledge of the state beef and milk industries by ADEPARÁ’s more experienced staff. In each region, a pre-established number of farms was randomly selected (first stage), and on each farm, a pre-set number of breeding females aged ≥24 months were randomly selected and tested for tuberculin (second stage) to classify the property as infected or non-infected with tuberculosis.

In order to identify the risk factors associated with TBT, the selected properties were subjected to a questionnaire, drawn up according to the literature. All the information generated was entered into a specific database which was analyzed at the Epidemiology and Biostatistics Laboratory of the Collaborating Center for Animal Health at FMVZ-USP.

### 2.2 Sample planning

In the first stage of sampling, the number of properties selected per region was estimated using the formula for simple random samples by Thrusfield and Christley (19), with the following parameters for an infinite population: estimated infected herd prevalence of 0.25, 0.95 confidence level, and 0.05 error. Calculations were performed using EpiTools (20).

In the second stage of sampling, the number of animals to be tested on each selected property was defined using the concept of herd sensitivity (hSE) and herd specificity (hSP), adopting individual sensitivity and specificity values for the tuberculin comparative cervical skin test of 0.800 and 0.995, respectively (21), and a within-herd animal prevalence of 0.20, in order to guarantee values of hSE ≥ 0.800 and hSP ≥ 0.900. Calculations using the EpiTools program (22) determined the following operating procedures: all animals on properties with up to 20 cows, 20 animals on properties with 21–99 cows, and 40 animals on properties with 100 or more cows. The cut-off limit for classifying a property as infected was one positive animal for those with up to 99 cows and two for those with 100 or more cows.

### 2.3 Tuberculin skin test

The comparative cervical tuberculin skin test was performed as recommended by the PNCEBT (6). Inconclusive animals were retested after 60 days; if the second test was inconclusive or positive, the animal was considered positive according to the Brazilian National Program (6). Cows within 30 days of calving were replaced with a new draw.

### 2.4 Calculation of prevalence

The apparent prevalence and confidence intervals were calculated according to the method described by Dean (23). The prevalence of positive animals and infected properties for the state and the prevalence of animals within the regions were weighted according to Dohoo et al. (24).

The weight of each property in the sample was given by (P1):


$$P1=\frac{\mathrm{Properties with reproductive activity in the region}}{\mathrm{Sampled properties with reproductive activity in the region}}$$

The weight of each animal in the sample was given by (P2):


$$\begin{array}{c} P2=\frac{\mathrm{Breeding females}\geq 24\;\mathrm{months in the property}}{\mathrm{Sampled breeding females}\geq 24\;\mathrm{months in the property}}\;\\ X\;\frac{\mathrm{Breeding females}\geq 24\;\mathrm{months in the region}}{\mathrm{Breeding females}\geq 24\;\mathrm{months in the sampled properties of the region}} \end{array}$$

Calculations were performed using R software (25).

### 2.5 Identification of risk factors

To Identify the risk factors associated with tuberculosis at herd level, an epidemiological questionnaire was administered to each sampled farm. Based on the literature and local characteristics, questions were asked about the type of farming enterprise and breeding, species raised on the property, herd size, type of milking, number of milkings, use of artificial insemination, presence of other domestic and wild species, diagnosis of tuberculosis, use of whey in calf feed, purchase of animals, and indirect contact between farms (sharing pastures or water troughs with another farm). Following the methodology proposed by Hosmer and Lameshow (26), an exploratory analysis of each variable was initially carried out using the chi-square or Fisher’s exact test, which were offered for multivariable logistic regression, using the stepwise forward method. The variables were entered into final multivariable regression model one by one, in increasing order of *p*-value, and remained in the model if they had a *p* ≤ 0.05, provided they did not remove statistical significance from the other variables. Calculations were performed using Python software (27).

### 2.6 Limitations of the study

This was a cross-sectional study with two-stage cluster sampling. Since we had a significant number of bTB-infected and uninfected properties, it was possible to study risk factors using a case–control approach. However, the definition of cases and controls was subject to misclassification based on the herd sensitivity and herd specificity defined for an intra-herd prevalence of 0.20 (hSE ≥ 0.800 and hSP ≥ 0.900).

## 3 Results

**Figure 2:**
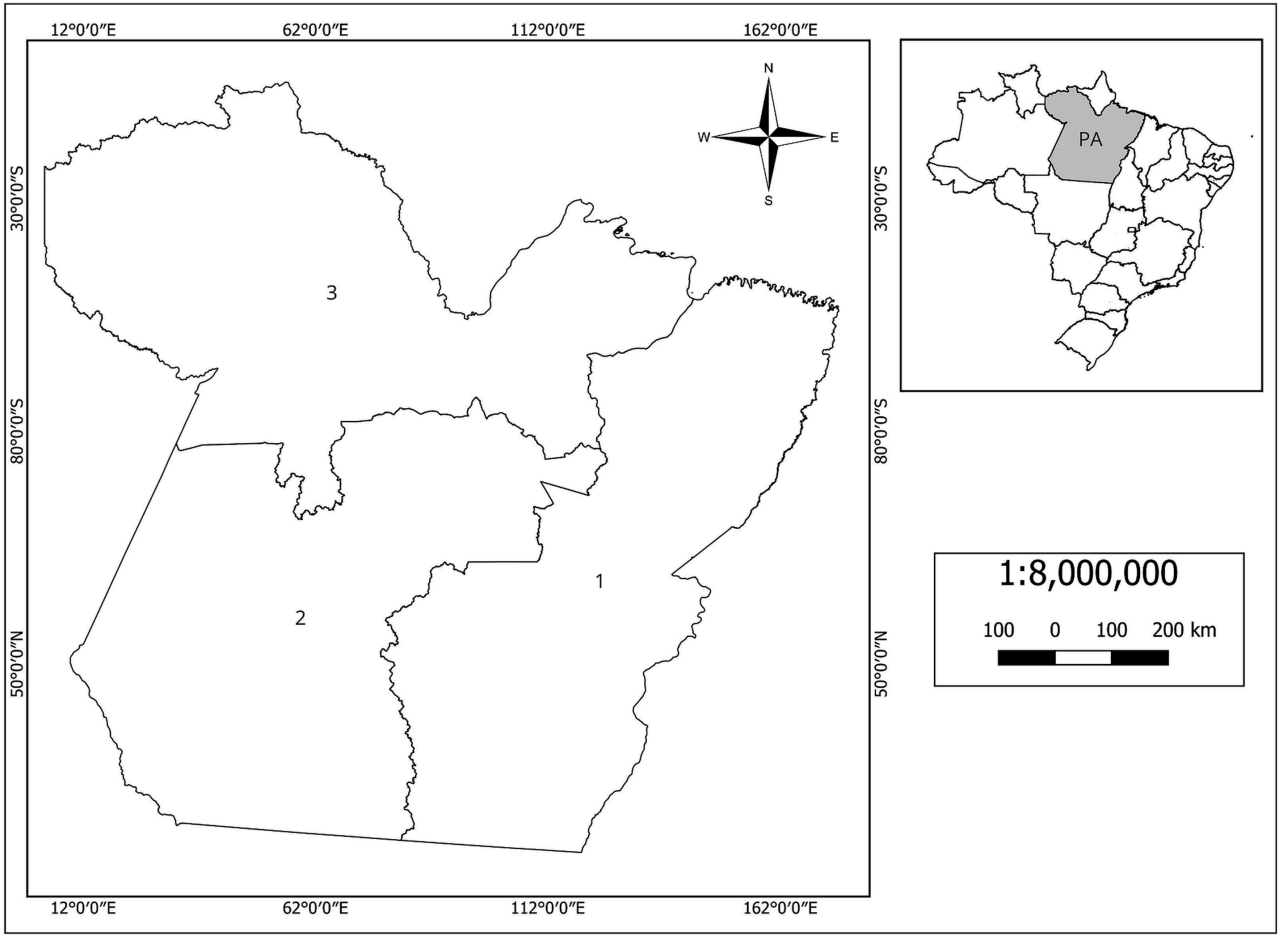
Map of the state of Pará divided into three regions for the study of bovine and bubaline tuberculosis. In detail, the location of the state in Brazil.

The state of Pará was divided into three regions (Figure 2): Region 1 is the most important for the beef and dairy industries, housing around 70% of all cattle in the state; Region 2 has only 22% of the state’s bovid population and is characterized by the presence of a vast area of protected lands; Region 3 is greatly influenced by the flood and ebb regimes, contains the Marajó archipelago and holds only around 8% of the bovid population in Pará, with a significant number of buffaloes.

**Figure 3:**
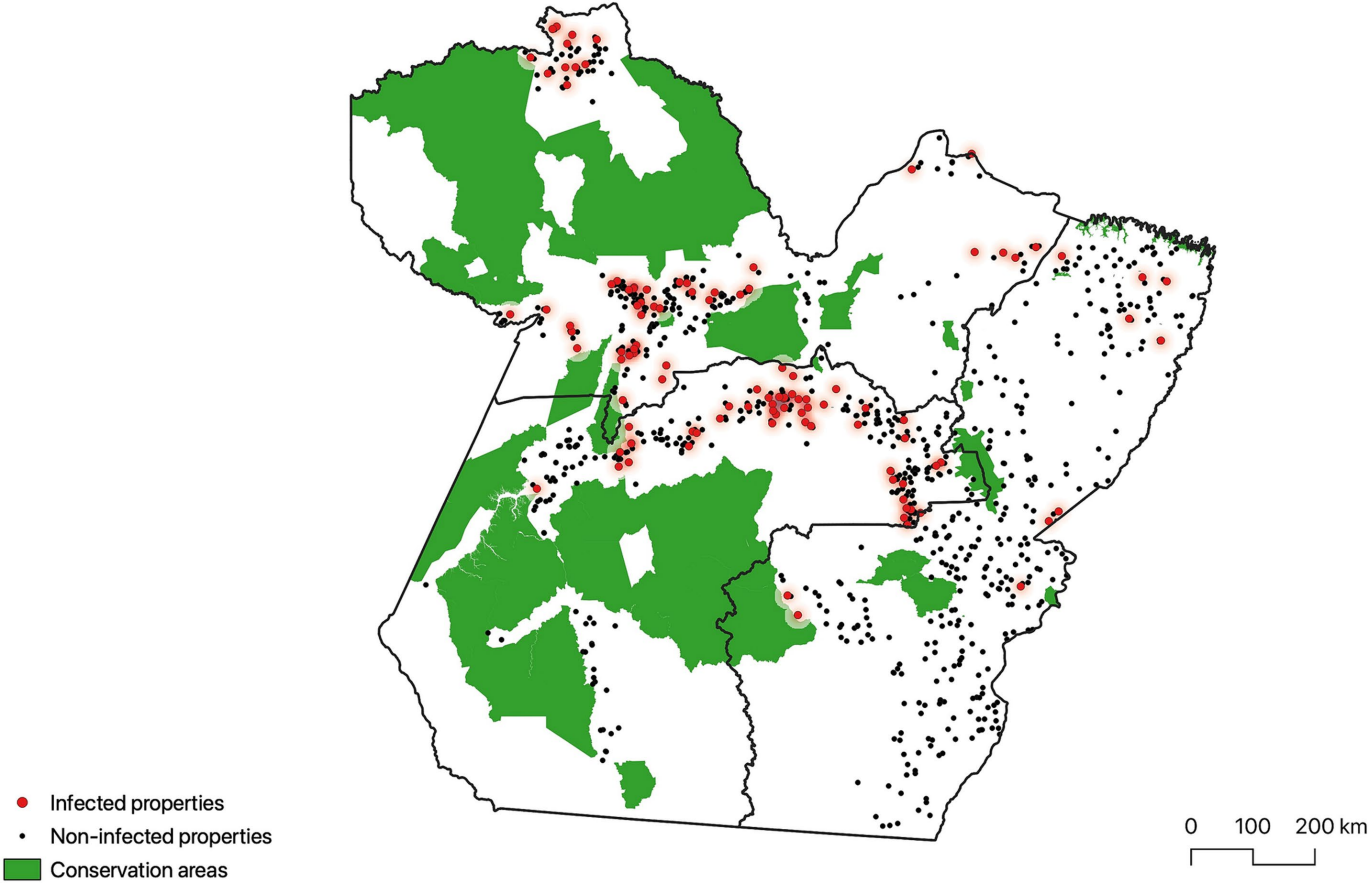
Map of the state of Pará, Brazil, showing the geographical location of the Conservation Areas and the properties sampled for the study of bovine and bubaline tuberculosis (infected and non-infected).

Table 1 presents demographic data on the state’s bovid population and the sample used in this study. Table 2 shows the prevalence of bTB at the farm and animal level. Figure 3 shows the geographical location and sanitary condition of the sampled properties.

**Table 1:** Registration and sample data for the bovine and bubaline tuberculosis study in the state of Pará, Brazil.

Region	Number of municipalities	State of Pará		Sample	
		Properties with reproductive activity	Breeding females aged ≥24 months	Properties with reproductive activity	Breeding females aged ≥24 months
1	96	59,661	6,157,313	355	6,749
2	16	24,521	1,947,681	304	6,105
3	32	14,702	679,469	317	4,286
Pará	144	98,884	8,784,463	976	17,140

**Table 2:** Prevalence of properties infected with tuberculosis and of bovines and buffaloes positive to the tuberculin test in the state of Pará, Brazil.

Region	Properties			Animals		
	Positive/tested	Prevalence	CI 95% (%)	Positive/tested	Prevalence	CI 95% (%)
1	11/355	3.1	1.3–4.9	21/6,749	0.24	0.09–0.40
2	49/304	16.1	12.0–20.3	113/6,105	1.7	1.1–2.2
3	59/317	18.6	14.3–22.9	188/4,286	4.8	2.4–7.3
Pará	119/976	8.6	7.1–10.0	322/17,140	0.91	0.68–1.00

We compare the prevalence of bTB-infected herds on beef, dairy, or mixed farms within the regions (Table 3). Looking at the central values for the prevalence, there is a tendency for the highest prevalence rates to be concentrated in dairy and mixed farms, except in Region 2, where the highest prevalence rates were found in beef and mixed farms, even with very close values. However, within regions no differences were detected between the prevalence of bTB-infected herds on beef, dairy and mixed farms, as the confidence intervals overlapped (Table 3). The small number of sampled dairy farms, especially in Region 3, resulted in the estimation of wide confidence intervals, increasing the likelihood of false-negative results in these comparisons.

**Table 3:** Prevalence of properties infected with bovine and bubaline tuberculosis in the state of Pará, Brazil, according to farming enterprise.

Region	Farming enterprise	Infected properties/tested properties	Prevalence of infected properties (%)	CI 95% (%)
1	Beef	3/156	1.9	0.66–5.5
	Dairy	1/37	2.7	0.49–13.8
	Mixed	7/157	4.5	2.2–8.9
2	Beef	28/165	17.0	12.0–23.4
	Dairy	2/25	8.0	2.2–25.0
	Mixed	19/110	17.3	11.4–25.4
3	Beef	38/233	16.3	12.1–21.6
	Dairy	4/11	36.4	12.4–68.4
	Mixed	16/70	22.9	14.6–34.0

Table 4 shows the prevalence of bTB-infected herds on farms that raised only cattle, only buffalo or both cattle and buffalo within regions.

**Table 4:** Prevalence of properties infected with bovine and bubaline tuberculosis in the state of Pará, Brazil, according to the species raised.

Region	Species raised on the property	Infected properties/tested properties	Prevalence of infected properties (%)	CI 95% (%)
1	Bovine	10/346	2,9	1.1–4.7
	Bubaline	1/3	33,3	0–86.7
	Bovine and bubaline	0/6	0,00	0–34.8^*^
2	Bovine	49/301	16,3	12.1–20.5
	Bubaline	0/0	0,00	-
	Bovine and bubaline	0/3	0,00	0–52.7^*^
3	Bovine	36/235	15,3	11.5–19.2
	Bubaline	17/57	29,8	18.0–41.7
	Bovine and bubaline	7/25	28,0	10.4–45.6

* Calculated by beta distribution and Monte Carlo simulation.

The register of properties in the state of Pará shows that 93.2% of them raise only cattle, 5.2% only buffalo, and 1.7% both buffalo and cattle. Only in Region 3 does buffalo farming take on greater importance, with 20% of properties raising only buffalo, 5% buffalo and cattle, and 75% only cattle. In regions 1 and 2, more than 98% of farms raise only cattle. Thus, the results of Table 4 indicate that buffalo make an important contribution to the high prevalence of bTB-infected farms exclusively in Region 3, which has the fewest properties and animals in the state (Table 1).

Table 5 presents the results of the univariate analysis and Table 6 the final multivariable logistic regression model for bovine and buffalo tuberculosis in the state of Pará.

**Table 5:** Results of the univariate analysis for the variables considered in the herd-level risk factors study for bovine and bubaline tuberculosis in the state of Pará, Brazil.

Variable	Number of properties		% of infected	*p* value
	Non-infected	infected		
Farming enterprise				0,8,761
Beef	485	69	14,2	
Dairy	66	7	10,6	
Mixed	295	42	14,2	
Type of breeding				0,2,221
Extensive	831	113	13,6	
Confined	9	4	44,4	
Semi-confined	1	0	0,0	
Species raised on the property				≤0,00001
Bovine	788	94	11,9	
Bubaline	42	18	42,9	
Bovine and bubaline	27	7	25,9	
Number of females aged ≥24 months				0,0236
Up to 69	655	79	12,1	
≥70 (3rd quartile)	202	40	19,8	
Number of milkings per day				0,2018
Do not milk	358	43	12,0	
1	305	40	13,1	
2 or 3	5	2	40,0	
Type of milking				0,0949
Do not milk	314	36	11,5	
Manual	300	37	12,3	
Mechanics at the foot	5	0	0,0	
Mechanics in the milking parlor	8	3	37,5	
Artificial insemination				0,0489
Do not use	793	103	13,0	
Use of artificial insemination and bull	21	7	33,3	
Use of artificial insemination only	6	0	0,0	
Presence of goat and sheep on the property				0,5,084
No	750	101	13,5	
Yes	107	18	16,8	
Presence of swine on the property				0,3,882
No	522	67	12,8	
Yes	335	52	15,5	
Presence of dog on the property				0,2045
No	192	20	10,4	
Yes	665	99	14,9	
Presence of cat on the property				0,5,519
No	468	69	14,7	
Yes	389	50	12,9	
Presence of wild Cervidae on the property				0,3,528
No	643	84	13,1	
Yes	214	35	16,4	
Presence of wild capybara on the property				0,625
No	341	44	12,9	
Yes	516	75	14,5	
Presence of wild marsupials on the property				0,625
No	140	18	12,9	
Yes	717	101	14,1	
Regular testing for tuberculosis				0,1,094
No	809	109	13,5	
Yes	39	10	25,6	
Use of whey in calf feed				0,0406
No	777	99	12,7	
Yes	5	1	20,0	
Purchasing of breeding animals in the last 12 months				0,0271
No	503	57	11,3	
Yes	325	60	18,5	
Renting pasture for cattle to graze and rest while being moved on foot				0,004
No	824	108	13,1	
Yes	5	4	80,0	
Sharing pasture with another property				0,1,084
No	708	90	12,7	
Yes	144	27	18,8	
Renting pasture to another property				0,2063
No	687	102	14,8	
Yes	157	17	10,8	
Sharing water troughs with another property				0,0013
No	669	76	11,4	
Yes	117	23	19,7	

**Table 6:** Final multivariable logistic regression model identifying the herd-level risk factors for bovine and bubaline tuberculosis in the state of Pará, Brazil.

Variable	Odds Ratio	CI 95%	*p* value
Species raised on the property			
Bovine (base category)			
Bubaline	3.80	2.03–7.13	≤0.0001
Bovine and bubaline	1.91	0.77–4.71	0.16
Number of breeding females aged ≥24 months on the property			
up to 69 (base category)			
≥ 70 (3rd quartile)	1.76	1.14–2.79	0.011
Renting pasture for cattle to graze and rest while being moved on foot			
No (base category)			
Yes	5.94	1.49–23.6	0.011

## 4 Discussion

Table 1 shows a large demographic difference between the regions studied, with region 1 holding approximately 3 times more animals than region 2 and around 9 times more than 3.

Regarding the prevalence of infected properties, the estimated value for the state does not adequately represent the situation observed in the regions (Table 2). There was no difference between regions 2 and 3, which presented higher values than region 1 (Table 2). A similar reasoning could be made regarding the prevalence in animals, except for the difference found among the three regions, composing the following decreasing scale of values: 3 > 2 > 1 (Table 2). These results show that division into regions captured intrastate heterogeneities.

In Brazil, the state of Pará had the same prevalence of infected properties as the states of São Paulo (9.0% [7.8; 10.5] (8)) and Espírito Santo (7.6% [5.7; 9.9], (28)), being the highest in the country (Table 2). The other 12 states studied had a lower prevalence of infected properties than Pará, which are: Tocantins (0.16% [0.02; 1.15], (7)), Federal District (0.36% [0. 0; 2.0], (29)), Santa Catarina (0.5% [0.07; 0.93], (30)), Mato Grosso do Sul (1.3% [0.7; 2, 4], (31)); Mato Grosso (1.3% [0.7; 2.4], (32)), Paraná (2.5% [1.9; 3.0], (3)), Rondônia (2.3% [1.5; 3.5], (33)), Rio Grande do Sul (2.8% [1.8; 4.0], (34)), Pernambuco (2.9% [1.8; 4.5], (35)), Goiás (3.4% [2.2; 4.7], (36)), Bahia (4.2% [3.1; 5.3], (37)) and Minas Gerais (4.3% [3.4; 5.2], (38)).

Among the 61 regions of the 14 Brazilian states already studied, only Regions 2 (11.2% [7.9; 15.5%]), 4 (11.8% [8.3; 16.6%]) 5 (13.9% [10.2; 18.8]) and 7 (13.3% [9.6; 18.1]) of São Paulo, located in the North of the state (Figure 1), which represent the traditional milk-producing area in Brazil (8), showed a prevalence of infected properties equal to that of Region 3 of Pará, the highest in the country (18.6% [14.3; 22.9], Table 2). Marajó Island is located in region 3, with large flooded areas and home to around 67% of the state’s buffaloes. In Region 3, properties that raised buffaloes alone or alongside cattle (a quarter of the farms) showed central estimates for the prevalence of bTB-infected herds higher than those observed for cattle-only farms (29.8, 28, and 15.3%, respectively, Table 4). Thus, buffalo farms make an important contribution to the high prevalence of infected herds in Region 3.

In Region 2, where the prevalence of infected farms is also high (16.1% [12; 20.3], Table 2), the disease is concentrated in cattle-only farms, as only 1.2% of the farms raise buffalo alone or alongside cattle.

Thus, our results indicate that buffalo make an important contribution to the high prevalence of infected farms exclusively in Region 3, which has the fewest properties and animals in the state (Table 1).

Although the methodology of the present study did not allow us to clarify the reasons underlying the higher prevalence on properties housing buffaloes, we hypothesize that the buffalo species’ gregarious behavior (39) increases the proximity between the animals and favors the transmission of *M. bovis*. In particular, on the Island of Marajó, where most of the state’s buffaloes are concentrated, the animals congregate in dry areas during the flood season and many of them walk freely, with no control over their movement, which also favors the transmission of *M. bovis* as well as other diseases.

The data in Table 3 show that within Regions 1 and 3, although a comparison of the confidence intervals does not reveal significant differences between the prevalence of infected herds on beef, dairy, and mixed farms, as they overlapped, the central estimates of these prevalences suggest a tendency for the disease to be concentrated preferentially on dairy and mixed ones. The association between bTB infection and milk production is very well documented in the international literature, including in Brazil (9, 40–43). On dairy farms, animals are grouped together daily for milking, which favors disease spread. This is especially true in Pará, where almost all properties extensively breed bovines and buffaloes.

The central estimates of these prevalences for the Region 2 suggest a tendency for the disease to be concentrated preferentially on beef and mixed farms, although the comparison of the confidence intervals does not reveal significant differences, as they overlapped (Table 3).

It’s important to note that the random sampling of properties showed that dairy farms are a clear minority in the state, especially in Region 3 (Table 4).

Our analysis revealed that species raised on the farm, properties size, and renting pasture for cattle to graze and rest while being moved on foot were risk factors increasing vulnerability to bovine and buffalo tuberculosis in the state of Pará (Table 6).

Farms that only raised buffalo were 3.8 times more likely to be infected with bTB than those that only raised cattle. As previously mentioned, we believe this is a consequence of the species’ highly gregarious behavior and the way it is raised in the state, especially in Region 3, where it represents one-fifth of the farms.

The size of a property, as measured by the number of adult breeding females, is a variable that has been commonly identified as a risk factor for slow-spreading endemic diseases. Owing to their size, large properties require more complex management practices, facilitating the spread of diseases among animals. Larger properties also have a greater demand for animal replacements, leading to more frequent purchases and introductions of animals, which is associated with increased vulnerability to tuberculosis (3, 8, 28, 32, 44, 45). As such, this variable indirectly suggests that the real risk factor for tuberculosis is the introduction of animals without taking precautions regarding tuberculosis. The purchase and introduction of animals is a classic risk factor for bTB, as described in the review on the topic by Skuce et al. (46). Further, this has been also verified in the Brazilian states of Rondônia and Minas Gerais (33, 38). As described previously, this purchasing involves the introduction of animals without any precautions against bTB.

Renting pastures for cattle to graze and rest while moving on foot is a unique practice still used in regions where moving cattle on foot is an economically viable option. This involves short-term rentals (ranging from one night to a few days), with a high turnover of animals from different flocks entering and leaving the pasture, increasing the risk of cattle on the property coming into contact with infected animals. This is the first time that this specific type of pasture rental has emerged as a risk factor in standardized studies conducted in Brazil to date. However, the traditional practice of renting pasture for animals from other properties has been identified as a risk factor for bTB in the states of São Paulo (OR = 1.58 [1.07; 2.33], (8)) and Pernambuco (OR = 5.12 [1.81; 14.45], (35)).

Although the study’s results on risk factors are biologically plausible and supported by scientific literature, the limitations mentioned in the Methods section suggest that a classic case–control study should be conducted to identify them with greater certainty.

In summary, despite cattle and buffalo farming being almost exclusively extensive in the state of Pará, the prevalence of properties infected with tuberculosis was surprisingly high, particularly in regions 2 and 3 (Table 2). This raises concerns regarding the zoonotic transmission of *M. bovis*, both occupationally and among populations that consume unpasteurized dairy products. Special attention should be paid to the traditional production of mozzarella with unpasteurized buffalo milk on the island of Marajó, located in Region 3, which can be authorized provided that the properties are accredited as free of tuberculosis and brucellosis. Otherwise, the milk produced by both species in the state must be pasteurized to give dairy consumers health guarantees.

In view of these results, we propose that the state of Pará, together with the private dairy and meat sectors, opt for the goal of controlling or eradicating the disease, as well as for mechanisms to verify its effectiveness.

In order to control the disease, these actors, led by the Official Veterinary Service, must develop policies to encourage the accreditation of tuberculosis-free properties, such as additional payment for milk and meat from free properties, and an efficient mechanism for replacing animals positive for diagnostic tests.

If eradication is targeted, the state must develop and implement a surveillance system (46) to detect infected properties in order to convert them into disease-free ones through routine indirect diagnostic tests, as recommended by PNCEBT (6). An important aspect of eradication would be to choose appropriate strategies for detecting infected properties, taking into account the organization and characteristics of the state’s milk and meat production chains. Ferreira Neto et al. (7) discussed the most appropriate strategies for detecting infected properties in the Brazilian environment, which included since tracing properties based on the condemnation of carcasses for tuberculosis in slaughterhouses, on tests carried out to move animals and also on the diagnosis of human tuberculosis in rural residents, until actively searching for infection on the dairy properties, on those with epidemiological link with infected ones, and also through cross-sectional studies in properties not reached by the above components.

According to Callefe and Ferreira Neto (46), given the complexity of a surveillance system, the ideal would be to start with a few components to detect infected properties, consolidate them, and only then insert more complex ones. Given the characteristics of the state, the following components could initially be implemented: tracing properties based on the condemnation of carcasses for tuberculosis in slaughterhouses; actively searching for infection on dairy and mixed farms, especially in confined breeding, on those that raise buffalo and also on those with epidemiological links to infected ones.

Regardless of the chosen objective, a health education program must be developed to inform Pará’s cattle and buffalo breeders that they need to test animals for tuberculosis before introducing them to their farms, as also avoid to rent pastures for cattle to graze and rest while moving on foot, as it is what contributed most to bTB spread in the state.

## Data Availability

The data sets analyzed during the current study are not publicly available because they belong to the Official Veterinary Service of the state of Pará (ADEPARÁ), but are available from the corresponding author on reasonable request.

## Supplementary material

Supplementary Data Sheet 1 Questionnaire applied to the sampled properties.

Supplementary Data Sheet 2 R and Python codes used in the analysis.
