# Supplementary material for: Epidemiological situation of bovine and bubaline tuberculosis in the state of Pará, Amazon region of Brazil
Source: Front Vet Sci. 2024 Nov 20;11:1466199. doi: 10.3389/fvets.2024.1466199 (PMC11618058; doi:10.3389/fvets.2024.1466199)
Supplement: Supplementary Data Sheet 1 — Questionnaire applied to the sampled properties. [file Data_Sheet_1.pdf]

TUBERCULOSE BOVINA E BUBALINA
Estudo Epidemiológico

01-Identificação:

Município: ________________________________Região N°: _______UF: _________

Proprietário: ____

Propriedade:____

Código de cadastro no serviço de defesa: _____________________________________

02 – Data das visitas:

Data da inoculação: _______/______/______

Data da leitura: _______/______/______

03 – Código do rebanho no estudo (10 dígitos)

|___|___|___|___|___|____|____|____|___|___|

04 – Coordenadas

Lat |____ |____| º|____|____|’ |____|____|.|____|”

Lon |____ |____| º|____|____|’ |____|____|.|____|”

05- Tipo da Exploração: ☐ corte ☐ leite ☐ mista
06- Tipo de Criação: ☐ confinado ☐ semi-confinado ☐ extensivo
07- Nº de Ordenhas por dia (apenas leite e mista): ☐ 1 ordenha ☐ 2 ou 3 ordenhas
08- Tipo de Ordenha (apenas leite e mista): ☐ manual ☐ mecânica ao pé ☐ mecânica em sala de ordenha
09- Produção de leite (apenas leite e mista): **a)** Nº de vacas em lactação: ________
b) Produção diária de leite na fazenda: ________ litros
10- Usa inseminação artificial? ☐ não ☐ usa inseminação artificial e touro ☐ usa só inseminação artificial
11- Raça predominante - Bovinos: ☐ zebu ☐ europeu de leite ☐ europeu de corte ☐ mestiço ☐ outras raças
- Bubalinos: ☐ murrah ☐ mediterrâneo ☐ carabao ☐ jaffarabadi ☐ outras raças

**12(a)- Bovinos existentes**									**12(b)- Bubalinos existentes**								
**Machos Castrados**	**Machos inteiros (meses)**				**Fêmeas (meses)**				**Machos Castrados**	**Machos inteiros (meses)**				**Fêmeas (meses)**			
Total	0-6	6-12	12-24	> 24	0-6	6-12	12-24	> 24 de reprod	Total	0-6	6-12	12-24	> 24	0-6	6-12	12-24	> 24 de reprod

13- Outras espécies na propriedade: ☐ ovinos/caprinos ☐ equídeos ☐ suínos ☐ aves comerciais ☐ cão ☐ gato
14- Espécies silvestres em vida livre na propriedade: ☐ não tem ☐ cervídeos ☐ capivaras ☐ marsupiais (gambá)
☐ outras:..
15- Tem pastagem que faz divisa com mata? ☐ não ☐ sim
16- Faz testes para diagnóstico de tuberculose? ☐ não ☐ sim
Regularidade dos testes: ☐ uma vez ao ano ☐ duas vezes ao ano ☐ quando compra animais
☐ quando exigido para trânsito/eventos/crédito
17- Nos últimos 2 anos houve aquisição de bovinos ou búfalos? ☐ não ☐ sim, **foram tuberculinizados** ( )sim ( ) não
Onde/de quem: ☐ em exposição ☐ em leilão/feira ☐ de comerciante de gado ☐ diretamente de outras fazendas
18- Local de abate das fêmeas e machos adultos no fim da vida reprodutiva:
☐ na própria fazenda ☐ em estabelecimento sem inspeção veterinária
☐ em estabelecimento de abate com inspeção veterinária ☐ não abate
19- Aluga pastos em alguma época do ano? ☐ não ☐ sim
20- Tem pastos em comum com outras propriedades? ☐ não ☐ sim
21- Compartilha outros itens com outras propriedades? ☐ não ☐ insumos ☐ equipamentos ☐ funcionários
22- Existem na propriedade áreas alagadiças às quais o gado tem acesso? ☐ não ☐ sim
23- A quem entrega leite (apenas leite e mista)? ☐ cooperativa ☐ laticínio ☐ direto ao consumidor ☐ não entrega
24- Resfriamento do leite (apenas leite e mista): ☐ não faz ☐ faz **Como:** em resfriador ou tanque de expansão próprio☐ coletivo☐
25- A entrega do leite é feita a granel (apenas leite e mista)? ☐ não ☐ sim
26- Produz queijo e/ou manteiga na propriedade? ☐ não ☐ sim, **finalidade:** ☐ p/ consumo próprio ☐ p/ venda
27- Consome leite cru? ☐ não ☐ sim
28-Tem assistência veterinária? ☐ não ☐ sim **De que tipo?** ☐ vet cooperativa\extensão ☐ veterinário particular
29-Alimenta bovinos com soro de leite bovino? ☐ não ☐ sim
30-Nos últimos 12 meses comprou bovinos/bubalinos: número de animais:............ , de quantas propriedades:..............
31-Nos últimos 12 meses vendeu bovinos/bubalinos: número de animais:............ , para quantas propriedades:.................
32- Compartilha aguadas\bebedouros com animais de outra(s) propriedade(s)? ☐ não ☐ sim
33- Propriedade possui área para pouso deboiada em trânsito? ☐ não ☐ sim
34- Classificação da propriedade? ☐ rural clássica ☐ aldeia indígena ☐ assentamento ☐ periferia urbana

NOME DO VETERINÁRIO:...ASSINATURA:...

35- INFORMAÇÕES SOBRE OS ANIMAIS TESTADOS				36- TUBERCULINA														
				TESTE							RETESTE							resultado pos: P neg: N
				aviária			bovina			ΔB-ΔA	aviária			bovina			ΔB-ΔA	
n.	identificação do animal/brinco	espécie bov:1 bub: 2	idade anos	A0	A72	A72-A0 (ΔA)	B0	B72	B72-B0 (ΔB)		A0	A72	A72-A0 (ΔA)	B0	B72	B72-B0 (ΔB)		
1																		
2																		
3																		
4																		
5																		
6																		
7																		
8																		
9																		
10																		
11																		
12																		
13																		
14																		
15																		
16																		
17																		
18																		
19																		
20																		

interpret. tub.	
ΔB – ΔA	resultado
≤ 1,9 mm	negativo
2,0 a 3,9 mm	inconclusivo
≥ 4,0 mm	positivo

CONTINUAÇÃO

35- INFORMAÇÕES SOBRE OS ANIMAIS TESTADOS				36 - TUBERCULINA														
				TESTE							RETESTE							resultado pos: P neg: N
n.	identificação do animal/brinco	espécie bov:1 bub: 2	idade anos	aviária			bovina			ΔB-ΔA	aviária			bovina			ΔB-ΔA	
				A0	A72	A72-A0 (ΔA)	B0	B72	B72-B0 (ΔB)		A0	A72	A72-A0 (ΔA)	B0	B72	B72-B0 (ΔB)		
21																		
22																		
23																		
24																		
25																		
26																		
27																		
28																		
29																		
30																		
31																		
32																		
33																		
34																		
35																		
36																		
37																		
38																		
39																		
40																		

interpret. tub.	
ΔB – ΔA	resultado
≤ 1,9 mm	negativo
2,0 a 3,9 mm	inconclusivo
≥ 4,0 mm	positivo
