# Supplementary material for: Epidemiological situation of bovine and bubaline tuberculosis in the state of Pará, Amazon region of Brazil
Source: Front Vet Sci. 2024 Nov 20;11:1466199. doi: 10.3389/fvets.2024.1466199 (PMC11618058; doi:10.3389/fvets.2024.1466199)
Supplement: Supplementary Data Sheet 2 — R and Python codes used in the analysis. [file Data_Sheet_2.pdf]

The first step is to organize two data bases with the following characteristics:

Table 1. To calculate prevalence of infected herds and perform risk factors study (File1.csv)

regions (V1)	ID sampled properties (V2)	properties classification (0:not infected;1:infected) (V3)	propertie weight in the sample (V4)	farm interprise (0:beef; 1: mixed; 2:dairy) (V5)	species raised (0:bovine; 1: bovine+buffalo; 2: buffalo) (V6)	V7	V8	V9	V10	Vn

Table 2: To calculate prevalence of positive animals in each region and for the state (File2.csv)

regions (V1)	ID sampled properties (V2)	ID tested animals (V3)	test result for animals (0:neg;1:pos) (V4)	animal weight in the sample (V5)

1) R codes to calculate prevalence

1.1) R code to calculate prevalence of infected herds for each region

```
# Install packages
install.packages(c("survey"))
library("survey")

# Read database (CSV file)
# Modify sep and dec according to your csv file
df<-read.csv(file = "File1.csv", header=TRUE, sep=";", dec=",")

# Convert text to float
df$V4 <- as.numeric(df$V4)

# Specifies study design
design<-svydesign(
  id = ~V2,
  data = df,
  weight = ~V4,
  strata = ~V1
)

# Calculates herd prevalence for TB
svyciprop(~I(V3==1), design, method="logit")
```

```
# Calculates prevalence by stratum
str_prev <- svyby(~I(V3==1), ~V1, design, svyciprop,
        method="logit")

# Display prevalence
str_prev

#Display confidence interval
confint(str_prev)
```

1.2) R code to calculate prevalence of infected herds for each region, according to farming enterprise

```
# Install packages
install.packages(c('dplyr','DescTools'))
library(dplyr)
library(DescTools)

# Read database (CSV file)
# Modify sep and dec according to your csv file
df3<-read.csv(file="File1.csv", header=TRUE, sep=";", dec=",")

# Calculate prevalence of infected herds for each region, according # to farming
enterprise
df3 %>%
  group_by(V1,V5) %>%
  summarise(
    count=n(),
    success=sum(V3),
    prop=BinomCI(success, count,
        conf.level = 0.95,
        method = "clopper-pearson")
  )
```

1.3) R code to calculate prevalence of infected herds for each region, according to the species raised

```
# Install packages
install.packages(c('dplyr','DescTools'))
library(dplyr)
library(DescTools)

# Read database (CSV file)
# Modify sep and dec according to your csv file
df3<-read.csv(file="File1.csv", header=TRUE, sep=";", dec=",")

# calculate prevalence of infected herds for each region, according # to the species
raised
df3 %>%
  group_by(V1,V6) %>%
  summarise(
    count=n(),
    success=sum(V3),
```

```
  prop=BinomCI(success, count,
        conf.level = 0.95,
        method = "clopper-pearson")
 )
```

1.4) R code to calculate prevalence of infected herds for the state

```
# Install packages
install.packages(c("survey"))
library("survey")

# Read database (CSV file)
# Modify sep and dec according to your csv file
df<-read.csv(file="File1.csv", header=TRUE, sep=";", dec=",")

# Convert text to float
df$V4 <- as.numeric(df$V4)

# Specifies study design
design<-svydesign(
  id = ~V2,
  data = df,
  weight = ~V4,
  strata = ~V1
)

# Calculates herd prevalence for TB
svyciprop(~I(V3==1), design, method="logit")
```

1.5) R code to calculate prevalence of positive animals for each region

```
# Load library
library("survey")

# Read database (CSV file)
# Modify sep and dec according to your csv file
df2 <- read.csv(file = "File2.csv", header=TRUE, sep=";", dec=",")

# Convert text to float
df$V5 <- as.numeric(df$V5)

# Specifies study design
design1<-svydesign(
  id = ~V2,
  data = df2,
  weight = ~V5,
  strata = ~V1
)

# Calculates animal prevalence for TB
svyciprop(~I(V4==1), design, method="logit")

# Calculates animal prevalence by stratum
str_an_prev <- svyby(~I(V4==1), ~V1, design, svyciprop,         method="logit")

# Display animal prevalence by stratum
```

```
str_an_prev

# Display confidence interval for animal prevalence by stratum
confint(str_an_prev)
```

1.6) R code to calculate prevalence of positive animals for the state

```
# Load library
library("survey")

# Read database (CSV file)
# Modify sep and dec according to your csv file
df2 <- read.csv(file = "File2.csv", header=TRUE, sep=";", dec=",")

# Convert text to float
df$V5 <- as.numeric(df$V5)

# Specifies study design
design1<-svydesign(
  id = ~V2,
  data = df2,
  weight = ~V5,
  strata = ~V1
)

# Calculates animal prevalence for TB
svyciprop(~I(V4==1), design, method="logit")
```

2) Python codes to perform the herd-level risk factors study

2.1) Python codes to perform the univariate analysis

```
# Import packages
import pandas as pd
import statsmodels.formula.api as sm
import numpy as np
from scipy.stats import chi2_contingency
pd.set_option('display.max_rows',None)
pd.set_option('display.max_columns',None)

# Read database (CSV file)
# Modify sep and decimal according to your csv file
df = pd.read_csv('./File1.csv', sep=';', decimal=',')

# Define list of variables
variables = [“V5”, “V6”, “V7”, “V8”, “V9”, “V10”, … , “Vn”]

# Looping for cross-tabulation and Chi-Squared calculation
for variable in variables:
    table = pd.crosstab(df[variable],
                        df[“V3”]).apply(lambda r: r/r.sum(),
                                        axis=1)
    table1 = pd.crosstab(df[variable], df[“V3”])
    chi, p, df, exp = chi2_contingency(table1)
```

```
print(f'\n{variable.upper()} (p={p:.4f})
\n\n{table1}\n{table}\n')
```

2.2) Python codes to define the final multivariable logistic regression model

As explained in the Methods chapter, considering the p values obtained by the univariate analysis, the variables were entered into final multivariable regression model one by one, in increasing order of p-value, and remained in it if they improved the adjustment, provided they did not remove statistical significance from the other variables. The Python code used was:

```
# Import packages
import pandas as pd
import statsmodels.formula.api as sm
import numpy as np
pd.set_option('display.max_rows',None)
pd.set_option('display.max_columns',None)

# Read database (CSV file)
# Modify sep and decimal according to your csv file
df = pd.read_csv('./File1.csv', sep=';', decimal=',')

# Define list of variables
variables = [“V5”, “V6”, “V7”, “V8”, “V9”, “V10”, … , “Vn”]

# Perform logistic regression
# This stage was carried out iteratively, checking the quality of
# the model's fit to decide whether it would remain or be removed
# from the model. Only one variable was included/removed at each
# step.
log_reg = sm.logit("V3 ~ V5 + V6 + … + Vn ", data=df).fit()

# Checking the results
log_reg.summary()

# Calculate Odds Ratios
odds_ratios = pd.DataFrame(
   {
      "OR": log_reg.params,
      "Lower CI": log_reg.conf_int()[0],
      "Upper CI": log_reg.conf_int()[1],
   }
)
odds_ratios = np.exp(odds_ratios)
print(odds_ratios)
```
